# Lipolytic Activity of a Carboxylesterase from Bumblebee (*Bombus ignitus*) Venom

**DOI:** 10.3390/toxins13040239

**Published:** 2021-03-26

**Authors:** Yijie Deng, Bo Yeon Kim, Kyeong Yong Lee, Hyung Joo Yoon, Hu Wan, Jianhong Li, Kwang Sik Lee, Byung Rae Jin

**Affiliations:** 1 College of Natural Resources and Life Science, Dong-A University, Busan 49315, Korea; dengyijie@naver.com (Y.D.); boyeon@dau.ac.kr (B.Y.K.); 2 Department of Agricultural Biology, National Academy of Agricultural Science, Wanju 55365, Korea; ultrataro@korea.kr (K.Y.L.); yoonhj1023@korea.kr (H.J.Y.); 3 College of Plant Science and Technology, Huazhong Agricultural University, Wuhan 430070, China; huwan@mail.hzau.edu.cn (H.W.); jianhl@mail.hzau.edu.cn (J.L.)

**Keywords:** *Bombus ignitus*, bumblebee, carboxylesterase, triglyceride, venom

## Abstract

Bee venom is a complex mixture composed of peptides, proteins with enzymatic properties, and low-molecular-weight compounds. Although the carboxylesterase in bee venom has been identified as an allergen, the enzyme’s role as a venom component has not been previously elucidated. Here, we show the lipolytic activity of a bumblebee (*Bombus ignitus*) venom carboxylesterase (BivCaE). The presence of BivCaE in the venom secreted by *B*. *ignitus* worker bees was confirmed using an anti-BivCaE antibody raised against a recombinant BivCaE protein produced in baculovirus-infected insect cells. The enzymatic activity of the recombinant BivCaE protein was optimal at 40 °C and pH 8.5. Recombinant BivCaE protein degrades triglycerides and exhibits high lipolytic activity toward long-chain triglycerides, defining the role of BivCaE as a lipolytic agent. Bee venom phospholipase A_2_ binds to mammalian cells and induces apoptosis, whereas BivCaE does not affect mammalian cells. Collectively, our data demonstrate that BivCaE functions as a lipolytic agent in bee venom, suggesting that BivCaE will be involved in distributing the venom via degradation of blood triglycerides.

## 1. Introduction

Carboxylesterases have a broad substrate specificity and are involved in energy homeostasis and lipid metabolism [1]. Carboxylesterases catalyze the hydrolysis of esters and thioesters, including lipids. Lipases catalyze the reactions of long-chain triacylglycerols, whereas carboxylesterases catalyze the hydrolysis of short-chain triacylglycerols [2,3]. Moreover, carboxylesterases have been demonstrated to play detoxifying roles against drugs and toxicants [1].

In honeybees, carboxylesterases reportedly mediate physiological activities owing to their involvement in the detoxification or metabolism of insecticides [4] as well as the processing of dietary nicotine [5] and regulation of oxidative stress [6]. In numerous insect species, carboxylesterases are identified as major enzymes responsible for detoxifying xenobiotics [4,5,7,8]. On the other hand, carboxylesterases are present in bee venom [9,10] and wasp venom [11].

Bee venom is a mixture of toxic components and functions as a defense against predator attacks while also having components with pharmacological and therapeutic value [12,13,14,15]. Thus, bee venom has been used in alternative therapeutic methods, such as in bee venom therapy (VT) [14] and bee venom immunotherapy (VIT) [16,17]. Various pharmacotherapeutic effects of bee venom have been reported in the treatment of several disorders, including arthritic rheumatism, pain, and cancer [13,14,15]. Furthermore, the proteomic analysis of honeybee and bumblebee venoms has unraveled components of bee venom, such as peptides, proteins with enzymatic properties, and low-molecular-weight compounds [9,10,18,19]. Many studies have determined the biological and pharmacological properties of bee venom components, such as enzymes and peptides [9,10,13,20,21,22,23]. Although carboxylesterases present in honeybee (*Apis mellifera*) venom and bumblebee (*Bombus terrestris*) venom were identified as allergens [9,10], the biological and toxicological characteristics of bee venom carboxylesterases remain incompletely understood.

In the present study, we show the lipolytic activity of a bumblebee (*Bombus ignitus*) venom carboxylesterase (BivCaE). Because phospholipase A_2_ (PLA_2_) in bee venom is a cytotoxic and lipolytic enzyme that cleaves the glycerol backbone of phospholipids [24,25], we hypothesized that the carboxylesterase in bee venom acts as a lipolytic agent that produces toxicological rather than beneficial outcomes. We investigated the lipolytic role of BivCaE against triglycerides because carboxylesterase degrades triglycerides [2,3].

## 2. Results

### 2.1. BivCaE Is a Bee Venom Component

**Figure 1:**
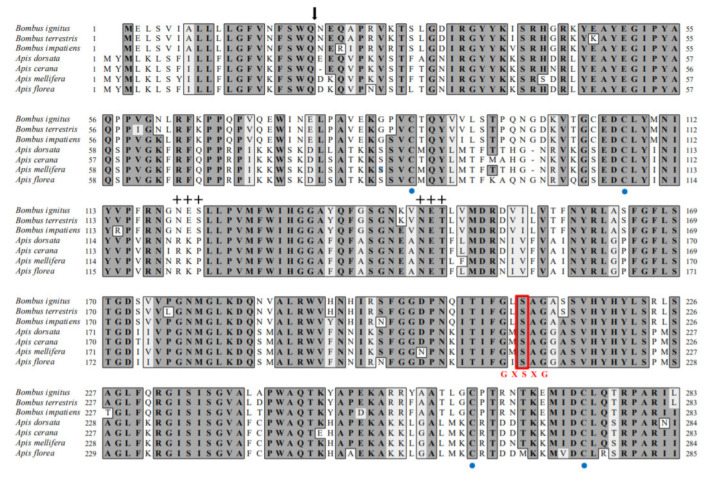

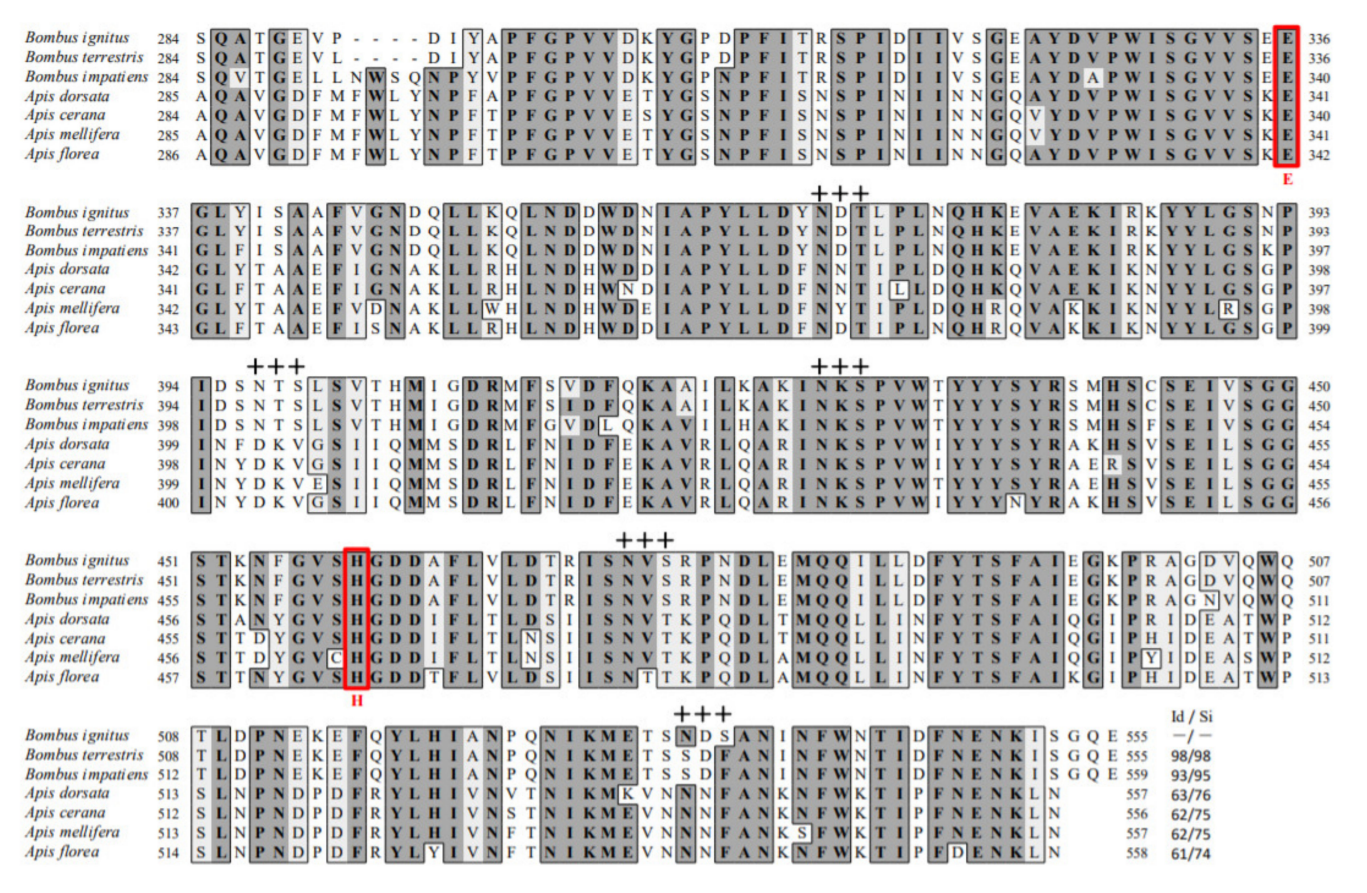
Alignment of the protein sequences of BivCaE and venom carboxylesterases from bee species. Predicted signal sequence (arrow), conserved cysteine residues (blue circles), and potential *N*-glycosylation sites (crosses) are indicated. The Ser–Glu–His catalytic triads and the classical GXSXG pentapeptide motif containing Ser residue are indicated with red letters. The protein sequences were aligned using BivCaE (this study, GenBank accession no. MW699017), *B. terrestris* venom carboxylesterase (XP_003394675), *B. impatiens* venom carboxylesterase (XP_012241172), *A. dorsata* venom carboxylesterase (XP_031367211), *A. cerana* venom carboxylesterase (XP_016912910), *A. mellifera* venom carboxylesterase (NP_001119716), and *A. florea* venom carboxylesterase (XP_031776417). Identity/similarity (Id/Si) values were determined using BivCaE sequence as a reference.

We cloned a BivCaE-encoding cDNA in *B. ignitus* worker bees in our search for identifying bee venom components. BivCaE consists of a predicted 19-amino acid signal peptide and a 536-amino acid mature protein (Figure 1). BivCaE exhibited high similarity to bumblebee venom carboxylesterases (93–98% protein sequence identity) and showed 61–63% protein sequence identity with honeybee venom carboxylesterases (Figure 1). Also, the protein sequence analysis of BivCaE revealed typical features of carboxylesterases, including a catalytic triad composed of Ser–Glu–His and a consensus active site motif GXSXG (Figure 1). These features indicated that BivCaE is a carboxylesterase.

**Figure 2:**
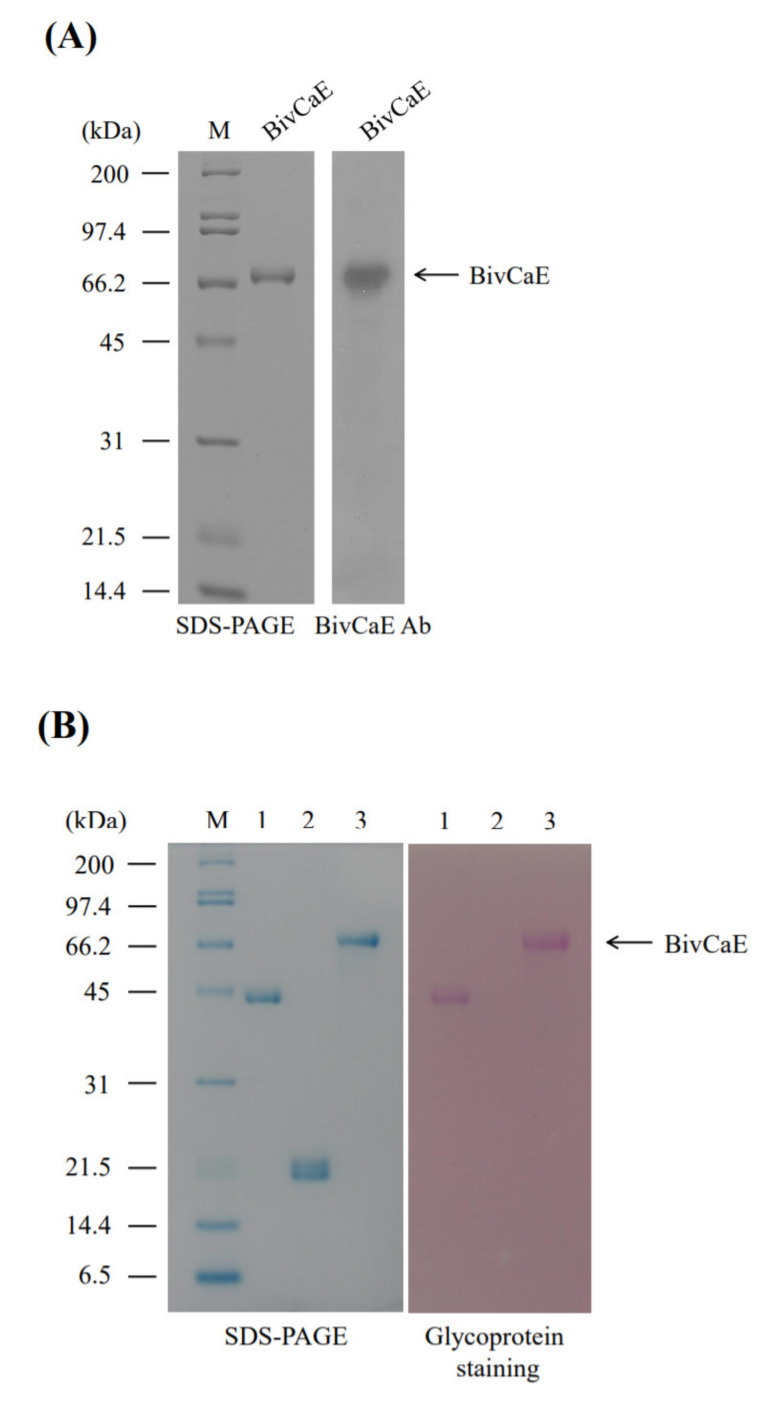
Production of recombinant BivCaE protein. (**A**) Recombinant BivCaE protein purified from baculovirus-infected insect cells was analyzed using 10% sodium dodecyl sulfate–polyacrylamide gel electrophoresis (SDS-PAGE) (**left**) and Western blot carried out by employing anti-BivCaE antibody against recombinant BivCaE (**right**). A molecular weight standard (M) is indicated. (**B**) Glycoprotein staining of recombinant BivCaE protein. Lane 1, horseradish peroxidase (positive control); lane 2, soybean trypsin inhibitor (negative control); and lane 3, recombinant BivCaE protein. The molecular weight standard (M) and recombinant BivCaE (arrow) are shown.

To characterize BivCaE, we produced recombinant BivCaE protein in baculovirus-infected insect cells and generated an anti-BivCaE antibody against recombinant BivCaE protein (Figure 2A). As shown in Figure 1, the protein sequence of BivCaE revealed several *N*-linked glycosylation sites. Glycoprotein staining analysis indicated that recombinant BivCaE protein was produced as a 71.4 kDa protein, which was 9.4 kDa greater than the predicted molecular mass of 62 kDa, thereby demonstrating that recombinant BivCaE protein was *N*-linked glycosylated (Figure 2B).

**Figure 3:**
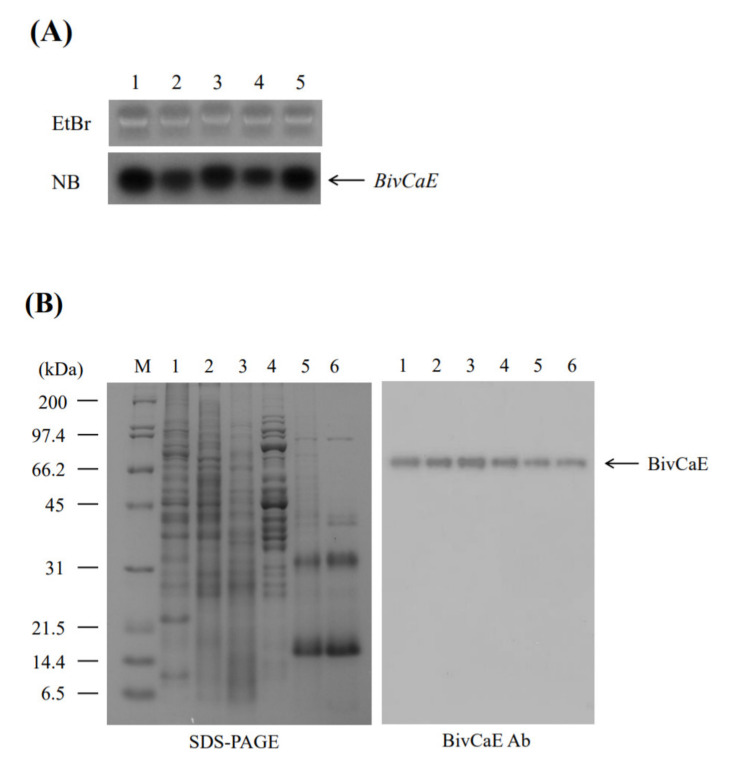
Expression of BivCaE in *B. ignitus*. (**A**) Expression of *BivCaE* in *B. ignitus* was carried out by Northern blot analysis (NB; lower panel) using total RNA from the epidermis (lane 1), the fat body (lane 2), the gut (lane 3), muscle (lane 4), and the venom gland (lane 5) of *B. ignitus* worker bees. The ethidium bromide-stained RNA gel (EtBr; upper panel) is shown. *BivCaE* transcripts are indicated. (**B**) Expression of BivCaE in *B. ignitus* was analyzed using 12% SDS-PAGE (left) and Western blotting technique by employing anti-BivCaE antibody (right). Protein samples were prepared from the epidermis (lane 1), the fat body (lane 2), the gut (lane 3), muscle (lane 4), the venom gland (lane 5), and the secreted venom (lane 6) of *B. ignitus* worker bees. The molecular weight standard (M) and BivCaE proteins (arrow) are shown.

The expression profile of BivCaE in *B. ignitus* worker bees was examined to confirm that BivCaE is a component of *B. ignitus* venom. Northern blot analysis revealed *BivCaE* transcripts in all the tissues investigated in this study (Figure 3A). Western blot analysis indicated that BivCaE proteins were detected in all the tissues, consistent with the Northern blot data, and demonstrated that BivCaE is present in the venom secreted by *B. ignitus* worker bees (Figure 3B); therefore, this result confirms that BivCaE is a venom component.

### 2.2. BivCaE Functions as a Carboxylesterase

**Figure 4:**
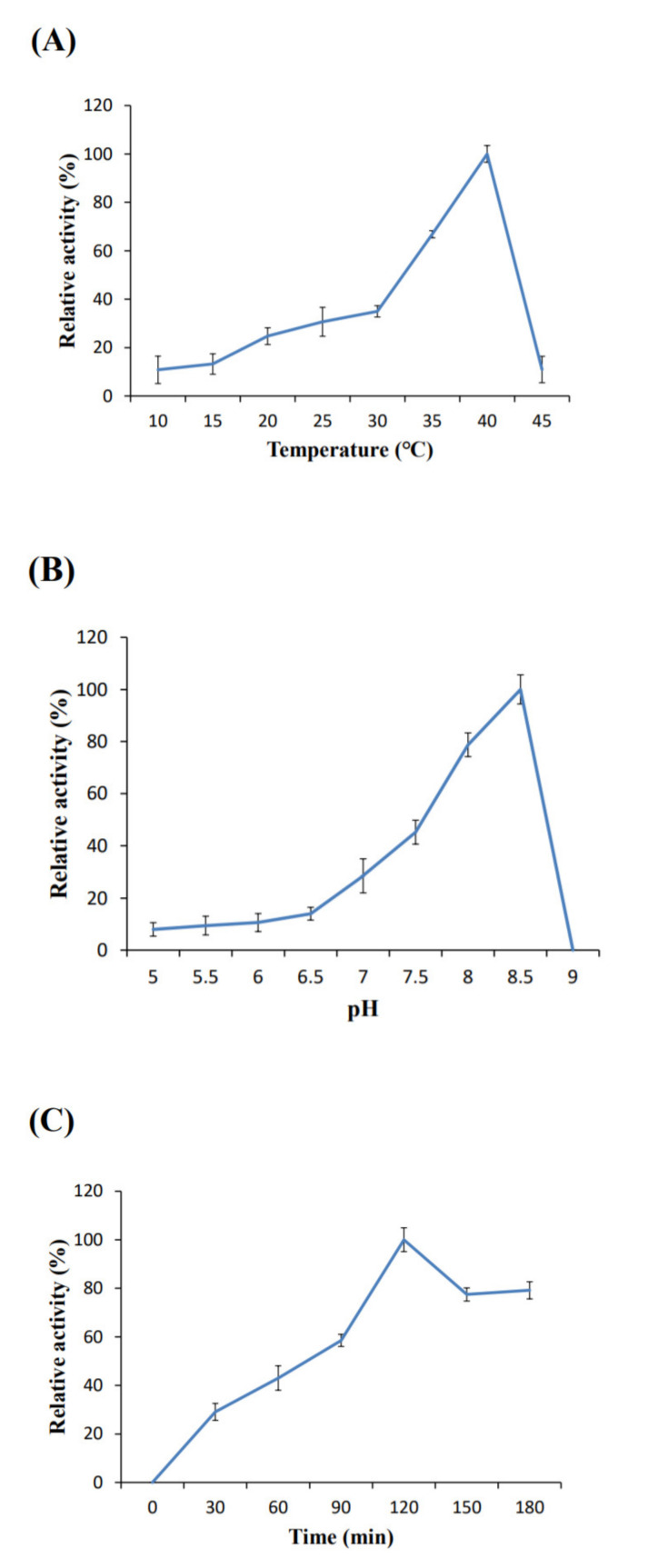
Enzymatic properties of recombinant BivCaE protein. Temperature (**A**), pH (**B**), and incubation time (**C**) for the optimum activity of recombinant BivCaE protein (*n* = 3). Error bars represent SD.

To assess BivCaE as a carboxylesterase, we assayed the enzymatic property of recombinant BivCaE protein. The enzyme activity of recombinant BivCaE protein was determined at varying pH levels, temperatures, and incubation times. When assayed under the condition of pH 8.5, the optimum temperature for the activity of recombinant BivCaE protein was 40 °C (Figure 4A). When assayed under conditions of 40 °C for 1 h, recombinant BivCaE protein showed the optimum activity at pH 8.5 (Figure 4B). The optimum incubation time for recombinant BivCaE activity was 120 min at 40 °C and pH 8.5 (Figure 4C). These results indicate that BivCaE is a carboxylesterase.

### 2.3. BivCaE Acts as a Lipolytic Agent

**Figure 5:**
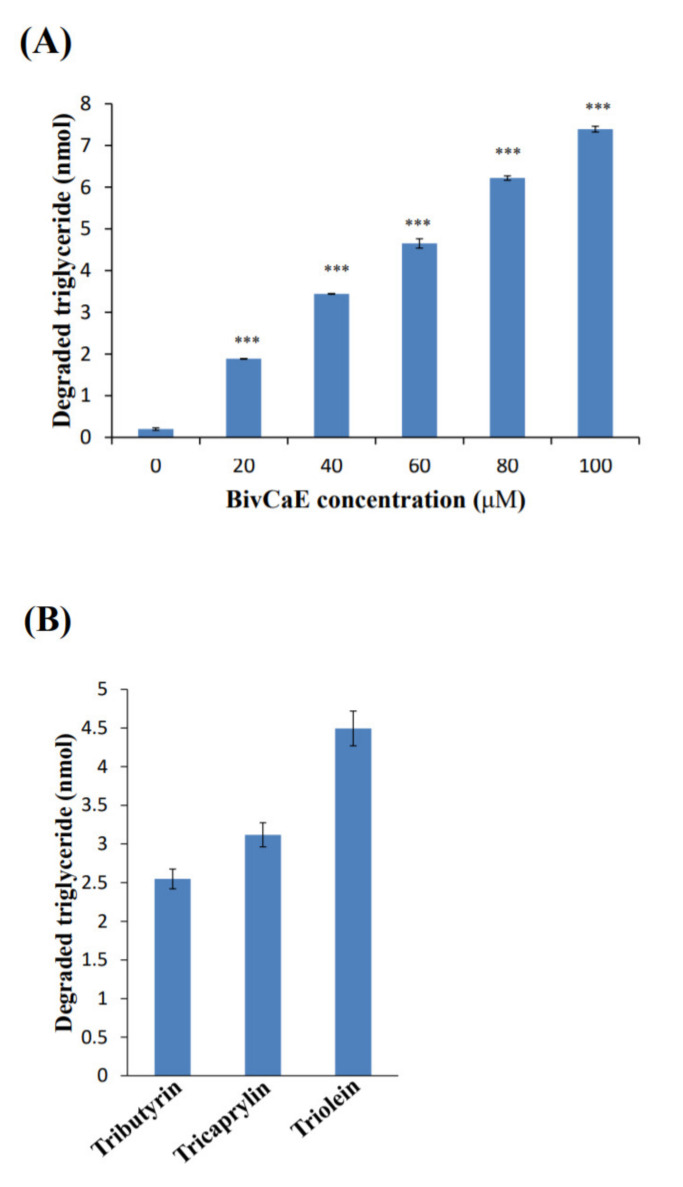
Lipolytic activity of recombinant BivCaE protein. (**A**) Degradation of triglyceride by recombinant BivCaE protein. Triglyceride was treated with recombinant BivCaE protein (0, 20, 40, 60, 80, or 100 μM) for 20 min (*n* = 3). Data are represented as the mean ± SD. *** *p* < 0.001. (**B**) Substrate specificity of recombinant BivCaE protein against triglycerides. Tributyrin (C4), tricaprylin (C8), and triolein (C18:1) were treated with recombinant BivCaE protein (60 μM) for 20 min (*n* = 3). Data are represented as the mean ± SD.

Because in this study, recombinant BivCaE protein revealed carboxylesterase activity, we first determined whether BivCaE exhibited lipolytic activity against triglycerides. The triglyceride degradation assay showed that recombinant BivCaE protein degraded triglycerides in a BivCaE concentration-dependent manner (Figure 5A). Next, the substrate specificity assay of BivCaE using tributyrin (C4), tricaprylin (C8), and triolein (C18:1) indicated that BivCaE exhibited high lipolytic activity against longer chains, such as tricaprylin and triolein (Figure 5B). These results support the fact that BivCaE in bumblebee venom acts as a lipolytic agent that degrades triglycerides.

**Figure 6:**
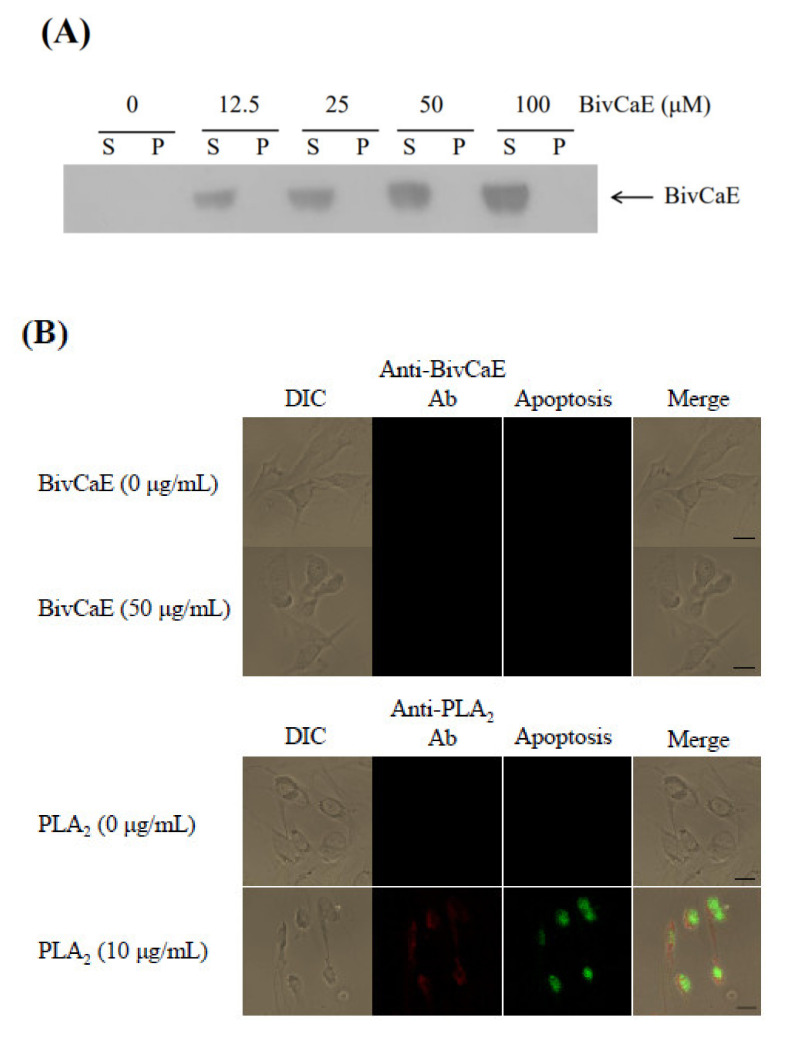
Cell binding assay of recombinant BivCaE protein. (**A**) Western blot analysis of the binding of recombinant BivCaE protein to mammalian cells. NIH 3T3 cells were incubated with recombinant BivCaE protein (0, 12.5, 25, 50, or 100 μg/mL of the medium) for 24 h. Recombinant BivCaE protein bound to the pellet (P) and free recombinant BivCaE protein in the supernatant (S) were analyzed using Western blotting by employing anti-His-tag antibody. (**B**) Representative immunofluorescence images of the recombinant BivCaE protein or *B. terrestris* PLA_2_ bound to mammalian cells. NIH 3T3 cells were incubated with recombinant BivCaE protein (0 or 50 μg/mL of the medium) or *B. terrestris* PLA_2_ (0 or 10 μg/mL of the medium) for 24 h. Apoptosis (green) and the binding of recombinant BivCaE protein (red) or *B. terrestris* PLA_2_ (red) to the cells were observed. Merged confocal images are shown. The scale bar is 20 µm.

As previously reported, the lipolytic enzyme PLA_2_ binds to mammalian cells and induces apoptosis [24]. Thus, to assess whether BivCaE affects the mammalian cell membrane, we investigated the recombinant BivCaE protein’s cell binding activity. A cell binding assay was performed using the Western blot technique, which indicated that recombinant BivCaE protein did not bind to mammalian cells (Figure 6A). We further investigated the binding of recombinant BivCaE to mammalian cells using immunofluorescence staining. We confirmed that recombinant BivCaE did not affect mammalian cells, whereas *B. terrestris* PLA_2_ bound to mammalian cells and induced apoptosis (Figure 6B), consistent with the findings of *B. ignitus* PLA_2_ [25]. Collectively, these results indicate that BivCaE in bumblebee venom can act as a lipolytic agent by degrading blood triglycerides but not whole mammalian cells.

## 3. Discussion

Bee venom is a complex mixture of bioactive substances, including various enzymes [9,10,13,14,26]. Considering that bee venom components may both facilitate venom distribution and cause tissue damage [9], the biological role of bee venom components is associated with toxicological outcomes but can also have pharmacological applications [13]. Bee venom’s pharmacotherapeutic effects have already been reported in treating several diseases, such as arthritic rheumatism, pain, and neoplastic diseases [13,14,15]. Moreover, bee venom proteomic studies have unraveled the venom composition and extended knowledge of potential venom allergens [9,10,18,19]. Although the bee venom carboxylesterase was identified as an allergen in honeybees and bumblebees [9,10], the enzyme’s functions as a bee venom component are not yet fully understood. Here, we reported the functional role of BivCaE as a bumblebee venom carboxylesterase.

We hypothesized that BivCaE is a *B. ignitus* venom carboxylesterase because it possesses a catalytic triad of Ser, Glu, and His residues and the classical GXSXG pentapeptide motif contains Ser residue, which are typical features of carboxylesterases [2,3,6]. Notably, the catalytic triad of Ser–Glu–His, a typical α/β hydrolase structure, and the consensus active site motif GXSXG were observed to be identical in bee venom carboxylesterases. Considering the protein sequence identity of BivCaE with putative venom carboxylesterases in other bee species, BivCaE is highly likely to be a *B. ignitus* bee venom carboxylesterase. Additionally, the expression profile obtained using Northern and Western blot analyses indicated that besides being a component of *B. ignitus* worker bee venom, BivCaE is present in many tissues, including the epidermis, the fat body, the gut, muscle, and the venom gland. The enzyme activity of BivCaE was confirmed using the recombinant BivCaE produced in insect cells. Together, these results indicate that BivCaE is a *B. ignitus* venom carboxylesterase.

Carboxylesterase, which belongs to the lipolytic enzyme family, exhibits lipolytic activity against triglycerides [2,3,6]. Because BivCaE is a bee venom carboxylesterase, we determined whether BivCaE also exhibits lipolytic activity and found that recombinant BivCaE degrades triglycerides and exhibits strong lipolytic activity against longer-chain triglycerides. Our data were similar to the results showing that carboxylesterases have a broad substrate specificity [1] and exhibit lipolytic activity against various triglycerides [2,3]. Collectively, our results show that BivCaE functions as a lipolytic agent, defining the role of BivCaE in *B. ignitus* venom.

Bee venom PLA_2_, known as a lipolytic enzyme, cleaves the glycerol backbone of phospholipids and induces cytotoxicity in mammalian cells [24,27]. Our present study revealed that purified *B. terrestris* PLA_2_ bound to mammalian cells and induced apoptosis, consistent with the findings of *B. ignitus* PLA_2_ in our previous study [25]; in contrast, recombinant BivCaE did not affect mammalian cells. Because bee venom is a mixture of toxic components that mediate the dissemination of venom and may also damage tissues [9], we also hypothesized that BivCaE might serve as a toxic component. Considering that bumblebee venom serine protease facilitates the distribution of venom throughout the bloodstream in mammals via fibrin(ogen)olytic activity [20,22], our results suggest that the lipolytic activity of BivCaE in bumblebee venom contributes toward the distribution of the venom components via degradation of blood triglycerides.

In conclusion, our data show evidence of the lipolytic activity of BivCaE against various triglycerides and of the role of BivCaE as a lipolytic agent in bumblebee venom. Together, our findings not only establish the biological and toxicological basis of bee venom carboxylesterase but may also offer insight into potential biomedical applications, including VT and VIT treatments.

## 4. Materials and Methods

### 4.1. Bumblebees and Venom Preparation

Bumblebee (*Bombus ignitus* and *B. terrestris*) worker bees were obtained from the National Academy of Agricultural Science, Republic of Korea. Venom from *B. ignitus* and *B. terrestris* worker bees was collected by stimulating their stings on the inside walls of collection tubes.

### 4.2. Complementary DNA (cDNA) Cloning and Sequence Analysis of BivCaE

The cDNA cloning of *BivCaE* cDNA was carried out using the *B. ignitus* cDNA library as previously described [20]. Plasmid DNA extraction was performed using the Wizard Mini-Prep Kit (Promega, Madison, WI, USA). The cDNA sequence was analyzed using the Basic Local Alignment Search Tool (BLAST) programs from National Center for Biotechnology Information (NCBI) [28]. The deduced protein sequences of BivCaE were aligned using MacVector (ver. 6.5, Oxford Molecular Ltd., Oxford, UK). The prediction of the signal sequence of BivCaE was carried out by the SignalP 4.1 Server [29]. The protein sequence of BivCaE was registered in GenBank (accession number MW699017).

### 4.3. Tissue Collection

Tissue samples of *B. ignitus* worker bees were collected using a stereo microscope (Zeiss, Jena, Germany) and washed with phosphate-buffered saline (PBS; 140 mM NaCl, 27 mM KCl, 8 mM Na_2_HPO_4_, 1.5 mM KH_2_PO_4_; pH 7.4). The fresh tissues were used for total RNA and protein sample preparation.

### 4.4. RNA Extraction and Northern Blot Analysis

Total RNA was extracted with a Total RNA Extraction Kit (Promega) and was separated in a 1.0% formaldehyde agarose gel (5 µg/lane). After electrophoresis, total RNA was transferred onto a nylon blotting membrane (Schleicher & Schuell, Dassel, Germany) and hybridized with *BivCaE* cDNA labeled with [α-^32^P]dCTP (Amersham Biosciences, Piscataway, NJ, USA). The cDNA labeling was performed using the Prime-It II Random Primer Labeling Kit (Stratagene, La Jolla, CA, USA). Hybridization and exposure were carried out using the method described by Choo et al. [20] and Yang et al. [30].

### 4.5. Production of Recombinant BivCaE Protein

The production of recombinant BivCaE proteins was performed using a baculovirus expression system [31]. The *BivCaE* cDNA, including His-tag sequence, was PCR-amplified using the following primer set: forward (1–18) 5′-ATGGAACTATCAGTTATC-3′ and reverse (1651–1668) 5′-TTACTCCTGCCCACTTAT-3′. The *BivCaE* cDNA was introduced into the baculovirus vector *pBacPAK8* (Clontech, Palo Alto, CA, USA) to construct the *pBacPAK8-BivCaE*. For generating BivCaE protein-expressing recombinant baculoviruses, *pBacPAK8-BivCaE* (500 ng) was co-transfected with baculoviral DNA (100 ng) into insect Sf9 cells (1.0–1.5 × 10^6^ cells/well of a 6-well plate) using Lipofectin transfection reagent (Gibco BRL, Gaithersburg, MD, USA). Recombinant BivCaE proteins in insect Sf9 cells were produced by infection of recombinant baculoviruses [30]. Recombinant BivCaE proteins were purified using the MagneHis^TM^ Protein Purification System (Promega). Concentrations of recombinant BivCaE proteins were analyzed using a Bio-Rad Protein Assay Kit (Bio-Rad, Hercules, CA, USA).

### 4.6. PLA_2_ Purification

*B. terrestris* PLA_2_ from *B. terrestris* venom was purified using size-exclusion column chromatography [25]. The conditions for chromatography were as follows: Elution was performed at a flow rate of 0.5 mL/min using a HiPrep 16/60 Sephacryl^TM^ S-100 column (Amersham Biosciences, Baie d’Urfé, PQ, Canada). The column was equilibrated with 0.05 M sodium phosphate buffer (pH 7.2) containing 0.15 M NaCl.

### 4.7. Polyclonal Antibody Production

A polyclonal antibody was produced in mice [32]. Purified recombinant BivCaE (5 μg) or purified *B. terrestris* PLA_2_ (5 μg) mixed with Freund’s complete adjuvant (Sigma, St. Louis, MO, USA) was injected into Institute of Cancer Research (ICR) mice (Samtako Bio Korea Co., Osan, Korea). At 1-week intervals, the mice were given two successive injections of recombinant BivCaE or *B. terrestris* PLA_2_ mixed with Freund’s incomplete adjuvant (Sigma). The last injection was administered using recombinant BivCaE protein (5 μg) or *B. terrestris* PLA_2_ (5 μg) without an adjuvant. Three days after the last injection, the collected blood was centrifuged at 15,000× *g* for 10 min at 4 °C. The serum was used for Western blot analysis and immunofluorescence staining.

### 4.8. Protein Analysis

Protein analysis was carried out using sodium dodecyl sulfate–polyacrylamide gel electrophoresis (SDS-PAGE) and Western blotting. Protein samples were subjected to 10% or 12% SDS-PAGE and were stained with Coomassie Brilliant Blue R-250 [33]. Western blot analysis was carried out using the enhanced chemiluminescence (ECL) Western blot system (Amersham Biosciences) with anti-BivCaE antibodies. Glycoprotein was stained using Gel Code Glycoprotein Staining Kit (Pierce, Rockford, IL, USA).

### 4.9. Enzymatic Assay

Esterase activity of recombinant BivCaE protein was assayed using the method described by Rejón et al. [34]. The effect of pH (5–9), temperature (10–45 °C), and time of incubation (30–180 min) was measured by the amount of *p*-nitrophenol (*p*-NP) formed from *p*-nitrophenyl butyrate (*p*-NPB) ester. Recombinant BivCaE protein (0.05 mol/L) was added to a solution of 880 μL of 50 mM Tris-HCl (pH 8.0), 100 μL of 0.4% (*v*/*v*) Triton X-100, and 10 μL of 1.76% *p*-NPB (*v*/*v* in acetonitrile), and the reaction mixture was incubated for 30 min at 30 °C. The amount of *p*-NP released was analyzed at 405 nm. The enzyme activity of recombinant BivCaE protein was represented as the % of control activity. The triglyceride degradation assay was performed using a Chemical Chemistry Analyzer (Bio-Rad) according to the manufacturer’s instructions. Recombinant BivCaE (0–100 μM) was incubated with 10 nmol triglyceride for 20 min, and the degraded triglycerides were measured. The enzyme substrate specificity was assayed using tributyrin (C4), tricaprylin (C8), and triolein (C18:1) as substrates [3]. Recombinant BivCaE (60 μM) was incubated with 10 nmol substrates for 20 min, and the degraded triglycerides were measured.

### 4.10. Cell Binding Assay

The cell binding assay for recombinant BivCaE proteins was carried out using the murine fibroblast cell line NIH 3T3 [32]. NIH 3T3 cells were cultured in Dulbecco’s modified Eagle’s medium (DMEM, Sigma) supplemented with 10% fetal bovine serum at 37 °C in a 5% CO_2_ atmosphere. The cells cultured onto 6-well plates (1 × 10^6^ cells/well) were incubated with serially diluted recombinant BivCaE protein (0, 12.5, 25, 50, or 100 μg per mL of the medium) for 24 h. After incubation, the media were collected by centrifugation at 1000× *g* for 5 min, whereas the cell pellets were resuspended in PBS after washing two times with PBS. Western blot analysis of cell binding of recombinant BivCaE protein was performed using the cell pellets and supernatant samples with the anti-BivCaE antibody.

### 4.11. Immunofluorescence Staining

NIH 3T3 cells were treated with recombinant BivCaE protein (50 μg per mL of the medium) or *B. terrestris* PLA_2_ (10 μg per mL of the medium) for 24 h. Subsequently, the NIH 3T3 cells were double-labeled with mouse anti-BivCaE or anti-PLA_2_ antibody using an in situ cell death detection kit (Roche Applied Science, Mannheim, Germany). The cell binding assays of the recombinant BivCaE or PLA_2_ on the NIH 3T3 cells were carried out by incubation with mouse anti-BivCaE or anti-PLA_2_ antibody (diluted 1:200 (*v*/*v*)). Subsequently, the NIH 3T3 cells were incubated with tetramethylrhodamine isothiocyanate-conjugated goat anti-mouse Immunoglobulin G (IgG; diluted 1:300 (*v*/*v*); Santa Cruz Biotech, Inc., Santa Cruz, CA, USA). Apoptosis assay was performed in NIH 3T3 cells by incubation with the Terminal deoxynucleotidyl transferase dUTP nick end labelling (TUNEL) reaction mixture, including terminal deoxynucleotidyl transferase (TdT) and fluorescein-conjugated dUTP. After incubation at 37 °C for 1 h, binding of the recombinant BivCaE or PLA_2_ on NIH 3T3 cell surfaces and apoptosis of NIH 3T3 cells were observed using laser-scanning confocal microscopy (Carl Zeiss LSM 510, Zeiss, Jena, Germany).

### 4.12. Statistical Analysis

Data were represented as the mean ± standard deviation (SD). All data were analyzed through independent unpaired two-tailed Student’s t-test using statistical software (SPSS 22.0, IBM, Chicago, IL, USA). Statistical significance was represented as *p* values (* *p* < 0.05, ** *p* < 0.01, and *** *p* < 0.001).

## Data Availability

Not applicable.
